# Assessment of the prophylactic activity and pharmacokinetic profile of oral tafenoquine compared to primaquine for inhibition of liver stage malaria infections

**DOI:** 10.1186/1475-2875-13-141

**Published:** 2014-04-14

**Authors:** Qigui Li, Michael O’Neil, Lisa Xie, Diana Caridha, Qiang Zeng, Jing Zhang, Brandon Pybus, Mark Hickman, Victor Melendez

**Affiliations:** 1 Division of Experimental Therapeutics, Walter Reed Army Institute of Research, 503 Robert Grant Avenue, Silver Spring, MD 20910, USA

**Keywords:** Prophylactic activity, Pharmacokinetic profile, Tafenoquine, Primaquine, Liver stage, Malaria infections, *In vivo* image system

## Abstract

**Background:**

As anti-malarial drug resistance escalates, new safe and effective medications are necessary to prevent and treat malaria infections. The US Army is developing tafenoquine (TQ), an analogue of primaquine (PQ), which is expected to be more effective in preventing malaria in deployed military personnel.

**Methods:**

To compare the prophylactic efficacy of TQ and PQ, a transgenic *Plasmodium berghei* parasite expressing the bioluminescent reporter protein luciferase was utilized to visualize and quantify parasite development in C57BL/6 albino mice treated with PQ and TQ in single or multiple regimens using a real-time *in vivo* imaging system (IVIS). As an additional endpoint, blood stage parasitaemia was monitored by flow cytometry. Comparative pharmacokinetic (PK) and liver distribution studies of oral and intravenous PQ and TQ were also performed.

**Results:**

Mice treated orally with three doses of TQ at 5 mg/kg three doses of PQ at 25 mg/kg demonstrated no bioluminescence liver signal and no blood stage parasitaemia was observed suggesting both drugs showed 100% causal activity at the doses tested. Single dose oral treatment with 5 mg TQ or 25 mg of PQ, however, yielded different results as only TQ treatment resulted in causal prophylaxis in *P. berghei* sporozoite-infected mice. TQ is highly effective for causal prophylaxis in mice at a minimal curative single oral dose of 5 mg/kg, which is a five-fold improvement in potency versus PQ. PK studies of the two drugs administered orally to mice showed that the absolute bioavailability of oral TQ was 3.5-fold higher than PQ, and the AUC of oral TQ was 94-fold higher than oral PQ. The elimination half-life of oral TQ in mice was 28 times longer than PQ, and the liver tissue distribution of TQ revealed an AUC that was 188-fold higher than PQ.

**Conclusions:**

The increased drug exposure levels and longer exposure time of oral TQ in the plasma and livers of mice highlight the lead quality attributes that explain the much improved efficacy of TQ when compared to PQ.

## Background

**Figure 1:**
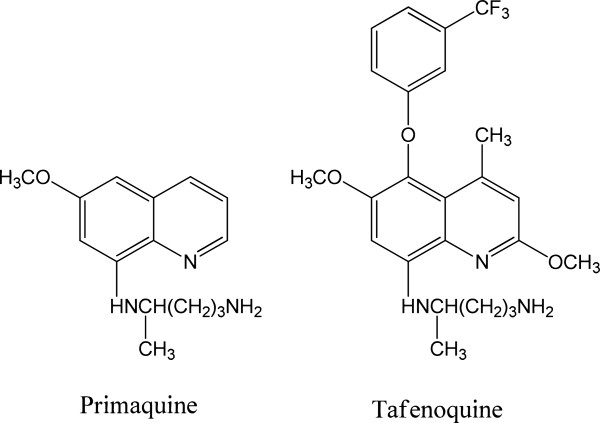
Chemical structures of primaquine and tafenoquine.

The 8-aminoquinoline (8-AQ) anti-malarials, such as primaquine (PQ), have attracted much interest as chemotherapeutic and prophylactic agents against the liver stages of *Plasmodium vivax* and *Plasmodium falciparum* malaria parasites (Figure 1). The 8-AQs are the only known class of drugs with activity against both *P. vivax* hypnozoites and *P. falciparum* gametocytes. The World Health Organization recommends PQ in combination with chloroquine for the radical cure of *P. vivax* malaria, although limited compliance with the 14-day dosing regimen is known to impact effectiveness [1]. The clinical value of this class is compromised by the toxic side effects, however, which include methemoglobinaemia and haemolytic anaemia in patients with deficiency in glucose-6-phosphate dehydrogenase (G6PD) activity [2].

Tafenoquine (TQ) is a 5-phenoxyl derivative of PQ with a long elimination half-life, which is under development by the US Army and GlaxoSmithKline Pharmaceuticals for treatment of relapsing *P. vivax* malaria and malaria prophylaxis. TQ has been shown to have activity against both the blood and liver stages of malaria. *In vitro* and *in vivo* animal models have shown that TQ is more potent and less toxic than PQ [3]. TQ has advantages as a chemoprophylactic agent, which is needed to address the problems of patient drug compliance, tolerance, and efficacy in both semi-immune and non-immune populations [4]. Phase I, II, and III clinical studies have shown that TQ is a safe, well-tolerated, and highly effective oral chemoprophylactic agent for the treatment of *Plasmodium* infections [5-7].

Given its long half-life of approximately 14 days, TQ has the potential to address problems with patient compliance during radical cure treatment of *P. vivax*, as Phase II studies of TQ treatment of vivax infections have shown that 1–3 days of treatment is effective to prevent *P. vivax* relapse when used alone or in combination with other anti-malarials [7,8]. In addition to effectiveness against hypnozoites, TQ also has been shown to have anti-gametocyte activity and long-acting blood stage activity against multidrug resistant strains [5]. These are valuable lead quality attributes that make TQ a potentially important agent for *P. falciparum* eradication [9]. Early animal studies have demonstrated that TQ had greater activity than PQ against the hypnozoite stages of *Plasmodium cynomolgi* and had equivalent efficacy against *Plasmodium knowlesi* liver stage parasites in rhesus monkeys [2]. Other studies in rhesus monkeys have shown that TQ is also more effective against the blood-stage asexual parasites of *P. cynomolgi, P. knowlesi* and *Plasmodium fragile* than PQ [10-12].

*In vitro* data have shown that TQ has from 4 to 100 times higher blood schizonticidal activity against cultured parasites of *Plasmodium berghei, Plasmodium yoelii*, and *P. falciparum*, including multi-drug-resistant strains [13-16]. TQ has been shown to inhibit sporozoite development in *Anopheles stephensi* mosquitoes that fed on *P. berghei* infected mice [17]. Further study in rodent malaria liver stage by real time *in vivo* imaging system (IVIS) have shown that the prophylactic activity of TQ is 3–4 times higher than PQ against liver stage malaria parasites administered subcutaneously [18]. The only study of the liver stage effects of TQ on a human *Plasmodium* species, *P. vivax*, demonstrated significant sporontocidal activity of TQ against *Anopheles dirus* mosquitoes infected with *P. vivax*[19]. An *in vivo* imaging of luciferase-expressing parasites was used to compare the liver stage activity of TQ versus PQ in rodents infected with *P. berghei* sporozoites. *In vivo* imaging of luciferase-expressing *P. berghei* parasites is conducted by using a sensitive camera to examine the luminescence emitted from anesthetized infected mice injected IP with luciferin. *In vivo* imaging has been successfully used to study *P. berghei*[18,20-25] and *P. yoelii*[26-28], which are commonly used to study the efficacy of agents against liver-stage. The authors’ laboratory acquired a luciferase-expressing *P. berghei* parasite from the MR4 repository, and this organism has used successfully to examine the time course of *P. berghei* infection in rodents, and the organ-specific activity associated with malaria drugs that exhibit true liver stage activity.

As previously discussed, TQ has been shown to be highly effective for both radical cure of relapsing malaria and causal prophylaxis. Relatively few mechanism studies have systematically characterized the sporontocidal property of oral TQ, however, compared to the current standard of care, oral PQ. Literature citations have relied on a single pharmacokinetic (PK) advantage of TQ’s longer half-life as a mechanism to explain enhanced liver stage activity. In this study, the liver stage activity of TQ and PQ in *P. berghei* infected mice was examined kinetically with a focus on prophylaxis of *P. berghei* sporozoites infected mice during the malaria liver-stage by using a modified real time imaging method. PK assays were used to provide a systematic PK profile for both drugs in mice to include 1) oral absolute bioavailability of TQ and PQ; 2) drug distribution in liver tissue, which is the target organ of both drugs; 3) drug elimination half-lives in liver and plasma; 4) drug concentration ratios of liver to plasma; and 5) total drug clearance.

## Methods

### Study drugs

TQ and PQ were formulated as a salt, but they were dosed in this study per the base compound weights. The bulk drug of TQ used for the test was synthesized for WRAIR by Ash Stevens, Inc. (Detroit, MI) and had a purity of > 99.8% by LC-MS/MS measurement.

### Sporozoites, inoculation and viability check

Luciferase-expressing *P. berghei* ANKA sporozoites were obtained from laboratory-reared female *An. stephensi* mosquitoes from the Department of Mosquito Biology, WRAIR. The mosquitoes were maintained at 18°C for 17 to 22 days after feeding on *P. berghei* malaria infected Swiss ICR mice. Salivary glands were extracted from malaria-infected mosquitoes and kept on ice in RPMI medium with 1% mouse serum. Sporozoites will be recovered by the method of Ozaki [29], and quantitated using a haemocytometer. Sporozoites isolated from the same batch of mosquitoes will be inoculated into C57BL/6 albino mice on the same day to control for biological variability in sporozoite preparations. Each C57BL/6 mouse will be inoculated intravenously in the tail vein with approximately 50,000 sporozoites suspended in 0.1 mL volume on day 0. To ensure that inoculated sporozoites are viable following the isolation procedure, they will be stained with a vital dye containing fluorescein diacetate (50 mg/mL in acetone) and ethidium bromide (20 μg/mL in phosphate-buffered saline; Sigma Chemical Co., St. Louis, Mo.) and counted in a haemocytometer. The viability of sporozoites ranged from 87 to 100%.

### Animals

Male 6-week-old C57BL/6 albino mice (NCI-Frederick, MD) were used for malaria liver-stage *in vivo* imaging assays, and male 7-week-old ICR mice (Charles River Lab, MA) were used for the PK evaluations. On arrival, the animals were acclimated for seven days in quarantine. The animals were housed in a cage maintained in a room with a temperature range of 64–79°F, 34–68% relative humidity and a 12-h light/dark cycles. Food and water were provided *ad libitum* during quarantine and throughout the study. The animals were fed a standard rodent maintenance diet. All animal studies were performed under IACUC approved protocols. These protocols detail the experimental procedures and designs as well as number of animals were used. All animal use, care, and handling were performed in accordance with the current “Guide for the Care and Use of Laboratory Animals” (8^th^ Edition, NIH, 2011) Science Education & Strategic Communications Walter Reed Army Institute of Research.

### Test agent administration and sampling

Novel and control anti-malarials were administered orally on days -1, 0, and 1 with respect to sporozoite inoculation. At 24, 48, and 72 hours post sporozoite infection, all inoculated mice were tested using the IVIS Spectrum instrument (Perkin Elmer, Hanover, MD). Additionally, blood stage infections were measured by flow cytometry. Positive and negative controls were used for the IVIS calibration in each test.

Causal prophylactic effects and delay in onset of parasitaemia in animals treated with TQ and PQ (Figure 1) were assessed in these experiments, which included a positive control, 4-methyl-PQ. All dosing was conducted based on the body weight at the time of preparation of the dosing solution. Both PQ and TQ were dissolved in distilled water and administered intragastrically with concentrations ranging from 1.25–25 mg/kg body weight. A once-a-day, three-consecutive-day treatment regimen (-1, 0, 1 day) was used in all assessments, on the day before sporozoite challenge, the day of challenge, and the day after challenge. Each animal received 0.1 - 0.2 ml of the oral solutions, delivered via an intragastric feeder (18-gauge) to the designated recipient. Stock solutions of test compounds were prepared fresh daily by grinding the needed amount of drug in cold (4°C) 0.5% (w/v) hydroxyethyl cellulose and 0.2% (v/v) Tween-80 (0.5% HECT) and diluting the resulting solution at variable concentrations. C57BL/6 albino mice were challenged intravenously with 50,000 luciferase-expressing *P. berghei* sporozoites extracted from the salivary glands of heavily infected *An. stephensi* mosquitoes. *In vivo* imaging was performed at 24, 48, and 72 hours after infection. At day 5–30 after infection, the same mice were analysed for blood stage infection by determination of the course of parasitaemia of tail blood by flow cytometry using an FC500 flow cytometer (Beckman-Coulter Co. CA). Blood samples (3 μL each) for parasitaemia determinations were collected on day 5 after inoculation and every other day thereafter until a pair of positive parasitaemia was demonstrated or for 30 days, then twice weekly for another 4 weeks if negative for parasites.

### *In vivo* image system (IVIS)

*In vivo* imaging studies of bioluminescence activity from luciferase expressing *P. berghei* infected mice were performed using a Perkin Elmer IVIS Spectrum (Hanover, MD). Mice were evaluated at 24, 48, and 72 hours post sporozoite inoculation to determine liver- and blood-stage malaria infection. Mice received 150 mg/kg luciferin (Gold Biotechnology, St. Louis, MO) intraperitoneally in a volume not to exceed 150 μL. Three minutes post luciferin administration the mice were anesthetized with inhaled isoflurane. The mice were then positioned ventral side up in the IVIS on a 37°C platform. The mice continue to receive isoflurane through nose cone delivery. The camera exposure times utilized were 1 and 5 minutes for the 24, 48, and 72 hour time points with f-stop = 1 and large binning setting. Quantitative analysis of bioluminescence emitted from whole bodies or region of intensity (ROI) were determined by measuring the luminescence signal intensity in photons/second using the ROI settings of the Living Image® 3.0 software. The ROI, which measurements were expressed in total flux of photons, was set to measure the abdominal area at the location of the liver from whole body imaging.

### Flow cytometry (FCM)

At day 5–30 after infection, the same mice were analysed for blood stage infections by quantitation of malaria parasites by flow cytometry (FCM). All FCM analyses were carried out with a FC500 MPL flow cytometer (Beckman Coulter, Fullerton, CA), which conducts five-colour analysis from either single or dual laser excitation. Infected erythrocytes, uninfected erythrocytes, and leukocytes were gated on logarithmic forward/side dot plots. Cells were analysed at an average rate of 2,000-3,000 erythrocytes/s. Filters were placed before the green (FL-1) and red (FL-2) photomultiplier tubes (PMTs) such that the green PMT registered fluorescence emission between 520 and 555 nm, and the red PMT measured emission greater than 580 nm.

A drop of blood (3 μL) from the mouse tail was collected directly into 0.3 ml of 1% heparinized isotonic buffer (PBS saline). In this study, 1 ml 0.04% of glutaraldehyde was used for fixation and the samples were then incubated at 4°C for 60 minutes. The cells were centrifuged at 450 × g for 5 min. The supernatant was removed by aspiration, and the cells were re-suspended in 0.5 ml PBS buffer supplemented with 0.25% (v/v) Triton X-100 for 10 minute incubation at room temperature. After centrifugation, the permeabilized cells were re-suspended in 0.5 ml of RNAse at 1 mg/ml concentrations and incubated for at least 2 hours at 37°C to ensure complete digestion of reticulocytes. *Plasmodium berghei* infection in mice results in anaemia which then results in reticulocytosis. Therefore, high RNAse concentrations for digesting large amounts of reticulocytes RNA were required for assessment of parasitaemia in this mouse model. YOYO-1 dye (from 1 mM stock solution in DMSO, as supplied by the manufacturer) was diluted to 2500 ng/mL (100-fold) concentrations in PBS and 20 μL of YOYO-1 solution at 2500 ng/mL was added to 0.5 mL of sample to a final dye concentration of 100 ng/ml of YOYO-1, which has been shown to be optimal to discriminate infected erythrocytes from the lowest (0.01%) to the highest parasitaemia counts (74.0%) [30,31].

### Pharmacokinetic (PK) studies

PK studies were performed using both intravenous (IV) and intragastric (IG) administration. For each time point to be acquired, three male ICR mice per time-point, aged seven weeks, were dosed at either PQ or TQ at 5 mg/kg (IV) or 20 mg/kg (IG). Drug vehicle for IV studies was 0.9% saline solution, and the formulated drugs were administered in a volume of 100 μL/20 g of body weight. For IG dosing, the drug vehicle was 0.5% HECT, administered at 100 μL/20 g. At each time point, plasma and liver samples were collected. Whole blood was collected by cardiac puncture. Blood samples were collected in lithium heparin tubes within 0 h (baseline) prior to drugs administration and at 0.5, 1, 2, 4, 8, 24, 48, 96, 168, and 336 h after drug administration. Following the separation of appropriate aliquots, plasma was obtained from the whole blood via centrifugation. All liquid and tissue samples were immediately preserved on dry ice and lately stored at -80°C until analytical work was performed.

### LC-MS/MS analysis

Sample was prepared by adding twice the normal volume of acetonitrile containing indomethacin internal standard (IS). The samples were mixed for 1 minute, centrifugation for 5 minutes, and the supernatant was transferred to an autosampler injection vial prior to separation by LC/MS/MS. Standard curve and quality control (QC) samples were generated by spiking interference free mouse plasma samples with known amounts of PQ, TQ and IS. Standard curve, QC, and assay samples were prepared and then 40 μL aliquots were injected into the LC/MS/MS system for chromatographic separation and subsequent mass spectrometric detection.

Blank liver homogenate was prepared by adding 5 mL of water for each gram of liver, then the mixture was homogenized using an ultra-sonication probe (VCX 750, Sonics & Materials, Inc., Newtown, CT). Plasma and liver homogenate standard curves were prepared via serial dilutions from a high concentration value (generally 500 ng/mL) through a series of 10–11 lower concentration values. The serial dilutions for analysis also included the preparation of 4–5 QC samples at a low range point and a high range point (generally 10 ng/mL and 100 ng/mL, respectively). Once the standard curve dilutions were prepared, a 100 μL aliquot was removed and extracted with 200 μL acetonitrile. The extracted samples were centrifuged at 10,000 rpm for 10 min and the supernatant was removed for analysis by LC-MS/MS. Sample drug concentrations were first interpolated from the standard curve, then multiplied by a factor of 6 to account for the drug levels present in the liver prior to dilution with water for homogenization.

Chromatography was performed using a Surveyor pump (Thermo Scientific, Waltham, MA) with Waters XTerra MS C18 50 mm × 2.1 mm id, 3.5 μm particle size columns (Waters Corp., Milford, MA). Mobile phase consisted of a water/0.1% formic acid (Solvent A)/acetonitrile/0.1% formic acid (Solvent B) gradient. The gradient began at 2% B, rose to 98% B from 1 min to 3.5 min, held steady for 2 min, then returned immediately to its starting composition and allowed to equilibrate for 1.5 min. Flow rate was 300 μL/min. Samples were injected using an HTC PAL autosampler (LEAP Technologies, Carrboro, NC). Tandem mass spectrometry was performed using a TSQ Quantum AM (Thermo Scientific).

### PK parameter determination

Drug concentrations were generated for each sample taken from animals dosed with both test drugs. A measured plasma and liver drug concentration *vs*. time curve was produced, in graphic and tabular form, for each subject on both linear/linear and log/linear scales, for the parent compound. Mean plasma drug concentration vs. time curves were also prepared separately. For the determination of initial approach to PK parameters of TQ and PQ in plasma and liver tissues after systemic application, a non-compartmental analysis was performed using Phoenix (version 6.1; Pharsight Corp., Mountain View, CA). Maximum plasma and liver concentration (*C*_max_), and time to maximum concentration (*t*_max_) of TQ and PQ were obtained from the plasma and liver drug concentration-time curves. The elimination half-life (*t*_1/2_) was calculated from In2/*k*_el_, which is the elimination rate constant calculated from the log concentration-time plot. The area under the curve (AUC) and the area under the first moment curve (AUMC) were determined by the linear trapezoidal rule with extrapolation to infinity based on the concentration of the last time point divided by the terminal rate constant. Mean clearance rate (CL) was determined by dividing the dose by the AUC_inf_ for intravenous injection for plasma samples. Absolute bioavailability was calculated by taking the dose-corrected area under the curve (AUC) from non-intravenous dosing divided by the AUC derived from intravenous dosing. Mean residence time (MRT) was determined by dividing the area under the first moment curve (AUMC) by AUC. The volume of the central compartment (Vz) and volume of the tissue compartment (Vz/F) were calculated as the product of CL and MRT.

### Data analysis

A Perkin Elmer *In Vivo* Imaging System (IVIS) Spectrum was used to provide a quantitative evaluation at 24, 48, and 72 hours post sporozoite inoculation to determine compound activity against liver- and blood-stage parasites. In the *in vivo* imaging experiments, causal prophylaxis activity, sporontocidal activity, parasite clearance, causal cure, delays in patency, and time to recrudescence were calculated as described previously [31]. The minimum curative dose (MCD_100_) in 100% animals was defined as the lowest dose, which cured all animals (>5 animals) in a group at any time during the first 30 days of the follow-up period. The data were generally found to fit a normal distribution. Means and standard deviations of photon measurement were calculated. Blood stage infection was defined as two positive blood samples by flow cytometry, taken daily apart. The negative blood samples were monitored until day 15 after inoculation. Coefficients of variation were calculated as a percentage by dividing the standard deviation by the mean value. Statistical analysis was conducted with Microsoft Excel software by using a Student t test for dependent samples to compare the means of paired and unpaired samples between treatment groups.

## Results

### Real time *in vivo* imaging of liver-stage model in mice

Real time *in vivo* imaging to determine the timing and level of luminescence measured from luciferase expression of sporozoites development in the liver was described previously by Ploemen *et al.*[18] and Thiberge *et al.*[20]. The IVIS instrument was used to measure luciferase activity of infected sporozoites in liver cells. Five mice per group were treated with or without different doses of prophylactic drugs starting one day before infection, the day during infection with luciferase-sporozoites, and the last dose at one day after infection. The test subjects were infected with 50,000 sporozoites by intravenous injection in the tail vein and the luminescence levels were determined 24, 48, and 72 hours post infection.

**Figure 2:**
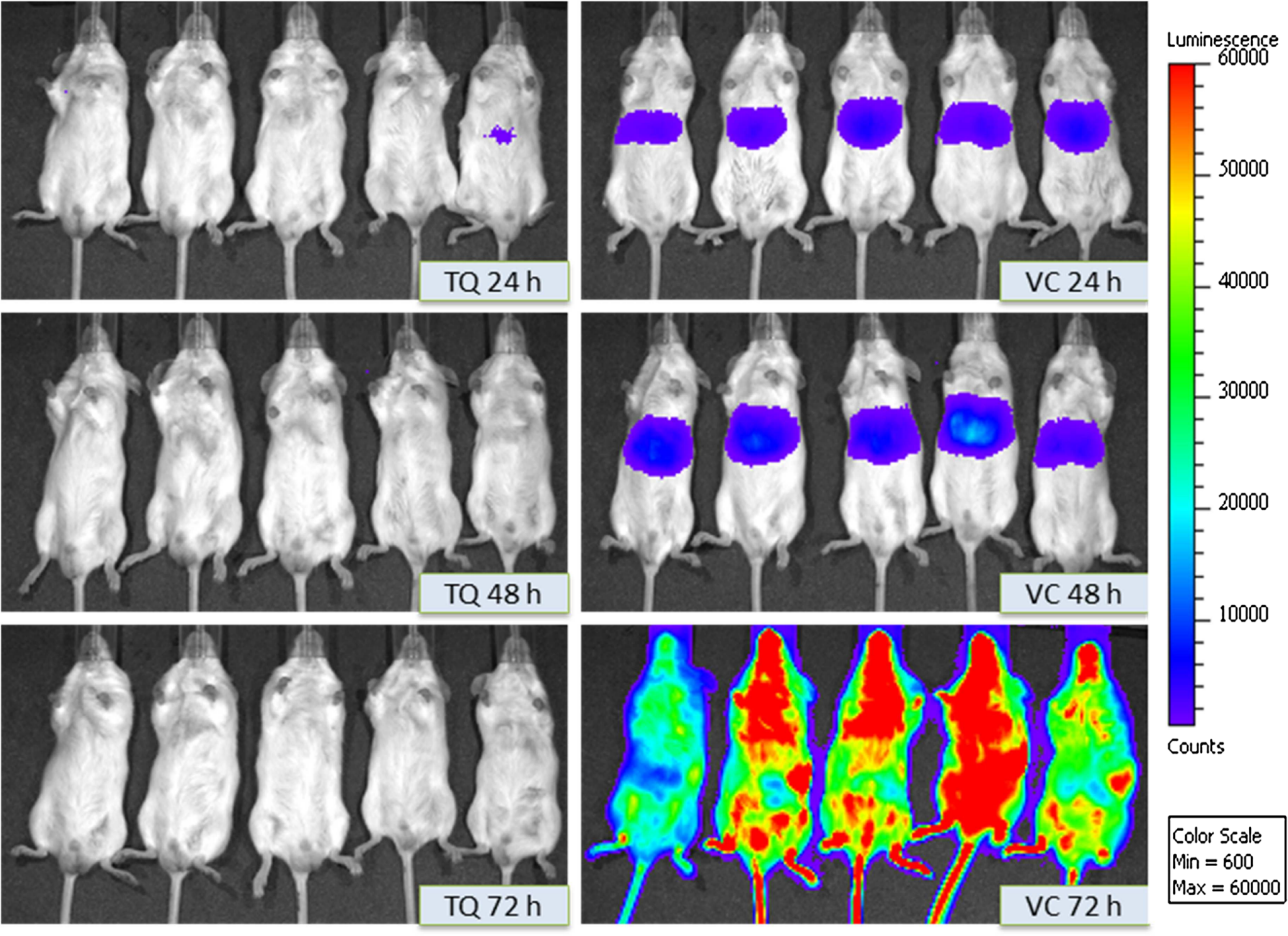
**Representative in vivo images (IVIS) of luminescence shown in the livers of live C57BL/6 albino mice at different time points after injection of 5 × 10**^**4**^**sporozoites.** Rainbow images show the relative levels of luminescence ranging from low (blue), to medium (green), to high (yellow/red). Luminescence levels (photons/sec) of livers in whole mice at 24, 48 and 72 hour time points following intragastric dosing daily for 3-consecutive-day on days -1, 0, 1, treated with tafenoquine (TQ) at a dose of 5 mg/kg (TQ, left) and vehicle control (VC, right) after sporozoite infection intravenously at day 0. Normally, *P. berghei* sporozoites reside in the mouse liver for 44–52 hours post-infection (n = 4).

Mean luminescence values (photon counts) collected from liver location of untreated control mice were 2.07 × 10^6^ photons/second (CV = 47.5%) at 24 hours and 35.48 × 10^6^ photons/second (CV = 44.9%) at 48 hours. In the untreated control animals, there was a strong increase in bioluminescence signal at 48 hours, and the liver region of intensity (ROI) was increased 17.2-fold when compared to 24 hours subjects. The liver stage of *P. berghei* is 48 hours in duration, and no significant blood stage activity was not observed after the 48 hour liver stage was completed (Figure 2, right). At 72 hours, mean luminescence signals of 127.67 × 10^6^ photons/second (CV = 65.3%), could be detected only in the whole bodies of untreated mice, which was 3.6 times higher than the luminescence measured at 48 hours, suggesting that the majority of the increase in luminescence observed was produced by blood stage *P. berghei* parasites [18]. This is consistent with the known 48-hour duration of the liver stage of *P. berghei* malaria model where parasites invade erythrocytes after the rupture of liver schizonts 48 hours post inoculation.

### Prophylactic efficacy of PQ and TQ after daily dosing for 3 days

Analysis of the inhibition of *in vivo* liver stage development by PQ and TQ was assessed by measuring luminescence using a Perkin Elmer Spectrum In *Vivo* Imaging System (IVIS). Mice were treated with daily doses of PQ at 5, 10, 15, 20, and 25 mg/kg body weight for 3 days and daily doses of TQ at 1.25, 2.5, 5, and 10 mg/kg body weight for three consecutive days (Table 1, Figure 2, left).

**Table 1:** **The causal prophylactic activities of tafenoquine (TQ) and primaquine (PQ) by using real time *in vivo *** **image system (IVIS) following single dose (-1 or 0 day after inoculation) or daily oral administrations for 3 days multiple doses (-1, 0, and 1 day after infection) against challenge with 50,000 sporozoites of the ANKA strain of *P. berghei *** **intravenously in male C57BL/6 albino mice with positive and negative controls (n = 5–20 each)**

**Test agents**	**Dose (mg/kg)**	**Oral dose**	**Date of dosing**	Suppression rate (%) IVIS^*^			Blood Infection by FCM^*^	**Number of C57BL/albino mice**			**Delay in patency (days after onset in controls)**	**Effects**
				**24 h**	**48 h**	**72 h**		**Challenged**	**Protected completely**	**Causal prophylaxis**		
Tafenoquine	10	3 days	-1, 0, 1	100	100	100	0/5	5	5	5/5	-	Full CP
	5	3 days	-1, 0, 1	90.4	100	100	0/13	13	13	13/13	-	Full CP
	2.5	3 days	-1, 0, 1	100	100	100	2/5	5	3	3/5	-	Partial CP
	1.25	3 days	-1, 0, 1	84.7	92.3	98.9	4/5	5	1	1/5	2,4,4,7	Suppression
	5	Single	-1	68.7	98.6	100	0/10	10	10	10/10	-	Full CP
	5	Single	0	66.0	91.1	98.5	4/5	5	1	1/5	4,2,4,9	Partial CP
Primaquine	25	3 days	-1, 0, 1	100	100	100	0/10	10	10	10/10	-	Full CP
	20	3 days	-1, 0, 1	100	100	100	1/10	10	9	9/10	6	Partial CP
	15	3 days	-1, 0, 1	100	100	100	4/20	20	16	16/20	2,2,2,1	Partial CP
	10	3 days	-1, 0, 1	100	97.6	100	5/10	10	5	5/10	2,4,2,2,4	Partial CP
	5	3 days	-1, 0, 1	82.4	77.5	89.4	5/5	5	0	0/5	2,2	Suppression
	25	Single	-1	45.7	0	0	5/5	5	0	0/5	-	Suppression
	25	Single	0	11.2	0	0	5/5	5	0	0/5	-	Suppression

^*^The infection was determined by recording the onset of IVIS for liver stage and parasitaemia for blood stage by using a flow cytometry system (FCM).

*CP* = causal prophylaxis.

In mice treated with 2.5 mg/kg body weight of TQ, 2 out of 5 mice showed a low level of luminescence ranging between 0.09 × 10^5^ and 0.35 × 10^5^ photons/second at 24–48 hours while the remaining 3 mice were negative. Two of these five mice developed a blood stage parasitaemia that was delayed by 2–4 days compared to the control mice. In 5 of 10 animals treated with 10 mg/kg PQ, a low level of luminescence was observed at 48 hours (0.66 × 10^5^ photons/sec) while the remaining 5 mice were negative. Five of these ten mice developed a blood stage parasitaemia that was delayed by 2–4 days compared to the control mice. All mice treated with 5 mg/kg of TQ and 25 mg/kg of PQ were luminescence negative during the observation period from 24 to 48 hours after infection, and none of these animals developed blood stage infection, suggesting that TQ is 5 times more potent for causal prophylaxis as PQ in this rodent malaria model. The complete inhibition of liver stage development by PQ at doses of 25 mg/kg body weight and higher and by TQ at doses of 5 mg/kg body weight and higher are in agreement with the inhibitory doses reported in the literature [12-15,18].

### Prophylactic efficacy of single doses of PQ and TQ

Mice treated with single oral doses of TQ at 5 mg/kg one day prior to infection showed causal prophylaxis results similar to those obtained through multiple doses of TQ at lower concentrations. A detectable luminescence signal in the liver was observed in mice treated with 5 mg/kg of TQ 24 and 48 hours after infection, but no blood stage parasitaemia was noted up to 30 days post infection suggesting that the suppressive effect of TQ against liver stage parasite growth and curative activity in the blood stage was due to a single oral dose of TQ (Table 1). In the group treated with a single oral dose of PQ at 25 mg/kg, high levels of luminescence and no suppression of signal were observed in the livers of mice after 24 hours suggesting incomplete inhibition of parasite growth (Table 1). In addition, analysis of these mice 5–30 days after infection showed severe parasitaemia in peripheral blood, suggesting that the single dose failure of PQ may be due to its short half-life.

### PK and bioavailability of intravenous and orally dosed PQ or TQ in mice

**Figure 3:**
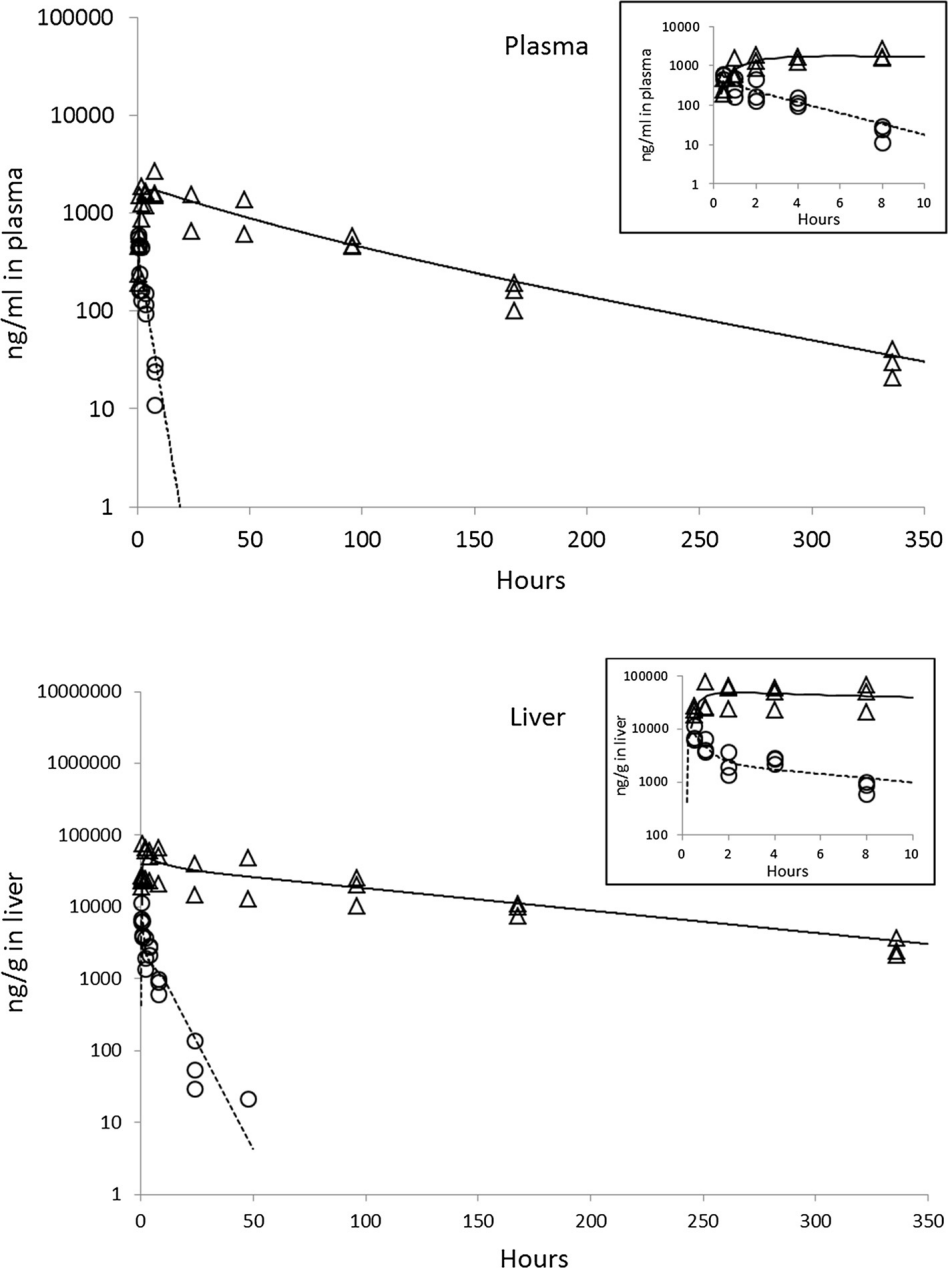
Individual plasma concentration-time profiles of tafenoquine (TQ, triangle markers) and primaquine (PQ, circle markers) measured by LC/MS/MS and computer fitted curves for TQ (solid line) and PQ (dashed line) at single intragastric dosage of 20 mg/kg in mouse plasma (top) and liver (bottom) (n = 3).

The individual computer fitted plasma concentration-time curves following single intragastric administration of PQ in mice are shown in Figure 3. The PK parameter estimates in plasma derived from animals dosed at 5 mg/kg PQ intravenously and 20 mg/kg intragastrically are summarized in Table 2. The mean C_max_ and AUC_inf_ of PQ was 1.21 μg/ml and 1.20 μg · h/ml, respectively, following single dose IV administration. The mean elimination half-life of intravenous PQ was 0.63 hours and the total clearance rate was 4.19 liter/h/kg.

**Table 2:** Pharmacokinetic comparison of tafenoquine (TQ) and primaquine (PQ) in plasma and liver tissue following single intravenous at dose of 5 mg/kg and intragastric at dose of 20 mg/kg administrations in male mice (n = 3)

**PK parameters**	**Tafenoquine (TQ)**		**Primaquine (PQ)**	
	**Plasma**	**Liver**	**Plasma**	**Liver**
Intravenous (5 mg/kg)				
C_max_ (μg/mL or g)	0.66 ± 0.13	44.76 ± 29.95	1.21 ± 0.21	3.99 ± 0.53
T_max_ (hours)	1.36 ± 1.11	3.69 ± 2.29	0.08 ± 0.0	0.39 ± 0.53
AUC_last_ (μg · h/mL or g)	29.63 ± 6.74	2493.82 ± 1361.10	1.20 ± 0.13	6.06 ± 1.39
AUC_infininty_ (μg · h/mL or g)	30.14 ± 6.65	2544.96 ± 1348.03	1.26 ± 0.14	6.14 ± 1.38
t_1/2_ elimination (h)	66.28 ± 10.32	70.02 ± 4.35	0.63 ± 0.08	1.32 ± 0.06
Vz (liter/kg)	16.76 ± 6.44	-	3.85 ± 0.71	-
Cl (liter/h/kg)	0.17 ± 0.04	-	4.19 ± 0.42	-
MRT (h)	55.92 ± 3.86	66.11 ± 19.19	0.68 ± 0.02	1.27 ± 0.11
AUC_inf_ ratio (liver/plasma)	-	87.24 ± 44.29	-	3.91 ± 0.54
Intragastric (20 mg/kg)				
C_max_ (μg/mL)	2.04 ± 0.59	56.90 ± 26.09	0.53 ± 0.07	8.15 ± 0.29
T_max_ (hours)	11.33 ± 5.37	1.17 ± 0.76	0.50 ± 0.0	0.50 ± 0.0
AUC_last_ (μg · h/mL)	137.91 ± 24.38	5140.52 ± 1794.60	1.26 ± 0.40	27.45 ± 1.84
AUC_infininty_ (μg · h/mL)	139.18 ± 24.92	5391.86 ± 1898.51	1.31 ± 0.41	27.67 ± 1.75
t_1/2_ elimination (h)	50.87 ± 4.23	75.48 ± 12.06	1.84 ± 0.45	4.28 ± 1.30
Vz (liter/kg)	10.60 ± 1.22	-	154.66 ± 61.83	-
Cl (liter/h/kg)	0.15 ± 0.03	-	57.94 ± 20.52	-
MRT (h)	62.08 ± 2.30	88.67 ± 12.98	2.28 ± 0.10	4.49 ± 0.25
AUC_inf_ ratio (liver/plasma)	-	48.91 ± 22.67	-	22.26 ± 6.73
Absolute bioavailability (%)	100.77 ± 37.49		28.32 ± 6.93	

*Vz* = Volume of distribution based on the terminal phase; *MRT* = mean residence time, *D* = Day.

Oral PQ showed very rapid absorption and reached a plasma peak concentration (T_max_) at 0.50 hours. The mean C_max_ and AUC_inf_ of PQ was 0.53 μg/ml and 1.31 μg · h/ml, respectively, following a single dose orally administered. The mean elimination half-life of oral PQ was shown to be 1.84 hours and the total clearance rate was calculated to be 57.94 liter/h/kg. The mean absolute bioavailability of oral PQ was calculated to be 28.32%, and this calculation was also based on the data derived from intravenous dosing.

The PK parameters assessed in animals after doses of 5 mg/kg TQ intravenously and 20 mg/kg intragastrically are summarized in Table 2. The mean C_max_ and AUC_inf_ of TQ was shown to be 0.66 μg/ml and 30.14 μg · h/ml, respectively, following IV administration. The mean elimination half-life of IV TQ was 66.28 hours and the total clearance rate was 0.17 liter/h/kg. TQ was shown to be absorbed very slowly with a T_max_ of 11.33 hours. The results demonstrated that the mean C_max_ and AUC_inf_ of TQ was 2.04 μg/ml and 139.18 μg · h/ml, respectively, following oral administration. The mean elimination half-life of TQ was 50.87 hours and the total clearance rate was 0.15 liter/h/kg. The mean absolute bioavailability of oral TQ was calculated to be 100.77% in mice, and this calculation was also derived from the data derived from intravenous dosing.

In comparison to the oral PK plasma parameters shown, oral TQ has an absorption rate that is 22.7-fold slower than PQ in mice. The C_max_ and AUC_inf_ of oral TQ were approximately 4-fold and 94-fold, respectively, higher than that of oral PQ. The elimination half-life of oral TQ was shown to be approximately 28-fold longer and the elimination clearance was shown to be 370-fold slower than oral PQ. The mean absolute bioavailability of oral TQ was 3.5-fold greater than oral PQ.

### Liver distribution of intravenous and oral PQ or TQ in mice

In this study, the parasite drug targets are inside hepatocytes and, therefore, the PK profile of PQ and TQ in the liver tissue is important. The liver tissue distribution of PQ and TQ was analysed, and the PK parameter estimates in the livers of mice dosed with PQ intravenously at 5 mg/kg and intragastrically with PQ at a dose of 20 mg/kg are summarized in Table 2. The mean C_max_ and AUC_inf_ of PQ was 3.99 μg/g and 6.14 μg · h/g, respectively, following a single IV administration of PQ. The ratio of the liver AUC to the plasma AUC in animals dosed with PQ intravenously was 3.91. The mean elimination half-life of PQ in the liver was 1.32 hours following IV injection. In the orally dosed PQ groups, the PK parameters derived showed a mean C_max_ and AUC_inf_ of 8.15 μg/g and 27.67 μg · h/g, respectively, following a single oral dose of PQ. The ratio of the liver AUC to the plasma AUC in animals dosed with PQ orally was 22.26. The mean elimination half-life of PQ in liver was shown to be 4.28 hours following oral dosing.

Following TQ administration, the PK parameters derived showed a mean C_max_ and AUC_inf_ of 44.76 μg/g and 2544.96 μg · h/g, respectively, following a single IV administration of TQ. The ratio of the liver AUC to the plasma AUC in animals dosed with TQ intravenously was 87.24. The mean elimination half-life of TQ in the liver was shown to be 70.02 hours following IV injection. After oral administration of TQ, the PK parameters derived showed a mean C_max_ and AUC_inf_ of 56.90 μg/g and 5391.86 μg · h/g, respectively, following single oral administration of TQ. The ratio of the liver AUC to the plasma AUC in animals dosed orally with TQ was 48.91. The mean elimination half-life of TQ in liver was shown to be 75.48 hours following oral dosing.

In comparison to the liver distribution of oral PQ, the C_max_ and AUC_inf_ of orally dosed TQ were approximate 7-fold and 195-fold, respectively, much higher than that of PQ in liver tissue. The elimination half-life of oral TQ was estimated to be 17.6-fold longer than the elimination half-life of oral PQ in the liver.

## Discussion

The screening and identification of agents that inhibit *Plasmodium* development in the liver is considerably more complex when compared to the very simple process of screening small molecules for inhibitory activity against blood-stage parasites. Quantitative analysis of liver stage development in small laboratory animals, *in vivo*, is hampered by the low levels of parasite infection as well as the complicated, time consuming and expensive traditional methods required to monitor parasite development, such as RT-PCR, RNA hybridization or direct counting of liver stages parasites. *In vivo* imaging is a technique that captures light emitted by the reaction of luciferase and its substrate luciferin, and this technique has been successfully used to study *P. berghei*[18,20-25] and *P. yoelii*[26-28] liver stage infections, which were commonly used to study the efficacy of agents against liver-stage *Plasmodium* parasites. The addition of flow cytometric monitoring of blood stage parasites using the YOYO-1 intercalating dye [30,31] provides a means of completely monitoring all aspects of the parasite life cycle in a rodent, from sporozoite infection to development of mature blood stage parasites.

To determine the timing and level of luminescence during *P. berghei* sporozoite development in the livers of infected rodents, groups of mice (n = 5) were infected intravenously with different numbers of sporozoites ranging from 5 × 10^3^ to 1 × 10^5^. Luciferase activity in the animals was visualized by whole body imaging using the IVIS Spectrum imaging system at 24, 48 and 72 hours after infection. In control, uninfected mice, luminescence values ranged between 1 × 10^2^ and 1 × 10^3^ photons/second. In mice infected with higher doses of sporozoites (5 × 10^4^ and 1 × 10^5^ sporozoites), all mice showed luminescence levels significant above background at 24 hours with luminescence activity of 2.1 × 10^6^ and 5.3 × 10^6^ photons/second, respectively. Mice infected with 2.5 × 10^4^ or fewer sporozoites showed a signal above background in only a few animals at 24 hours. In all infected mice there was a strong increase (6 – 17 fold) in bioluminescence signal at 48 hours. Therefore, 5 × 10^4^ sporozoites is the minimum sporozoite infectious burden required to induce luminescence levels above background in 100% of the C57BL/6 albino mice tested. After 48 hours, luminescence signals could be detected in the whole body of infected animals, resulting from parasites that had invaded erythrocytes after the rupture of the liver schizonts. Based on these observations, a window of luminescence intensities between 24 and 48 hours was determined in subsequent experiments in mice infected with a constant number of 5 × 10^4^*P. berghei* sporozoites to test for liver-stage inhibition of drugs.

*In vivo* assessment of drug efficacy against liver stage *Plasmodium* parasites through *in vivo* imaging offers clear advantages over standard methods of RT-PCR analysis of dissected livers or through analysis of the dynamics of blood stage infections examined subsequent to liver-stage infection. *In vivo* imaging is simple and rapid and allows, within the same animal, measurement of both the specific inhibition of liver stage development by a small molecule and subsequent effects on blood stage parasites. *In vivo* imaging analysis does not require sacrificing experimental animals and thereby reduces the number of animals required for experimentation since multiple measurements can be made in the same animal over time. Furthermore, *in vivo* imaging also has the advantage that it minimizes the biological variation within the study [24,28] as the *in vivo* analysis of PQ and TQ liver stage efficacy was performed with mice that were infected by intravenous injection of sporozoites. All the control mice in these experiments (i.e. infections in the absence of drug) injected intravenously with sporozoites showed strong luminescence signals at 24 and 48 hours after infection. Complete inhibition of signal in animals treated with PQ and TQ was observed at intragastric doses of 25 mg/kg and 5 mg/kg, respectively, which correspond to liver-stage inhibitory concentrations reported in the literature for primaquine and tafenoquine treated subcutaneously with drug [18]. In conclusion, quantitative analysis of liver stage development by real-time imaging should greatly aid the development of drugs that act against the liver stages of *Plasmodium* parasites*.*

PQ and TQ themself are generally believed unlikely to be the clinically active form of the drug, because they have similar multiple pathways and are extensively metabolized and less active than some of its metabolites or derivatives in *in vitro* models [16,32]. Determination of metabolic pathways *in vivo* that lead to efficacy is difficult, because there is no *in vitro* model of hypnozoites within liver cells with which to evaluate efficacy. Therefore, currently, the liver-stage efficacy was likely relating to the PK concentrations of the parent drug of PQ and TQ. The absorption rate (T_max_) of 11.33 hours observed in animals treated with TQ suggests a slow absorption, which implies a prolonged period of absorption in the gut. Dissolution studies of TQ in simulated gastric fluid demonstrate complete dissolution within 30 minutes [33]. Thus, the long apparent absorption phase may be due to a distal GI absorption site combined with slow clearance of TQ as T_max_ is a function of both absorption and elimination rates. PQ is well absorbed from the gastrointestinal tract with peak plasma levels attained within 5 minutes in mice. TQ was found to be slowly but extensively metabolized [33]. TQ has been shown to be eliminated via biliary excretion with enterohepatic recirculation, but it is not eliminated in the urine. PQ is similarly extensively and rapidly metabolized with less than 1% of the dose excreted as unchanged drug in the urine [33]. In this study, the clearance (CL) of TQ was only 0.15 L/hours/kg (3% of the hepatic blood flow), while PQ has a much more rapid clearance of 57.94 L/hours/kg.

TQ has a 3.5-fold higher bioavailability was observed following oral administration than oral PQ. Also, TQ has a half-life that is more than 28 times longer was noted than PQ. Figure 3 illustrates the effect of this difference in half-lives between these two 8-aminoquinolines. A single 20 mg/kg dose of TQ results in easily quantifiable concentrations for more than two weeks. However, a single 20 mg/kg dose of PQ results in no detectable concentrations within 1 day after dosing. Modeling of TQ, on the other hand, is best described by a two-compartment model. This kinetic difference results in more prolonged, high concentrations of TQ in the blood. These properties of TQ provide an advantage over PQ in that they may permit long-term dosing for prophylaxis and short-term or single dose therapy for terminal eradication or radical cure of *P. vivax* malaria, which will likely result in improved compliance and enhanced effectiveness.

In this study, TQ was found to be 5-fold more potent than PQ as a liver schizonticides. This may be explained in part by its full bioavailability, accumulation in the blood and hepatocytes, prolonged elimination, and its longer half-life, in addition to difference in intrinsic activity. PQ, on the other hand, does not accumulate inside blood and liver cells [33]. The increased drug exposure levels and longer exposure time of oral TQ in the plasma and livers of mice highlight the reasons for its enhanced anti-malarial activity.

## Competing interests

The authors declare that they have no competing interests.

## Authors’ contributions

QL and MO conceived of the study. LX, DC, QZ, and JZ conducted the liver-stage efficacy study with IVIS. BP and VM conducted LC/MS/MS study. MH reviewed and edited this manuscript. All authors read and approved the final manuscript.
